# Clinical application of transcranial magnetic stimulation in multiple sclerosis

**DOI:** 10.3389/fimmu.2022.902658

**Published:** 2022-09-05

**Authors:** Xiaoliang Zhou, Kailin Li, Si Chen, Wenbin Zhou, Jing Li, Qing Huang, Tingting Xu, Zhiyuan Gao, Dongyu Wang, Shuo Zhao, Hao Dong

**Affiliations:** ^1^ Department of Neurology, Xiangya Hospital, Central South University, Changsha, China; ^2^ National Clinical Research Center for Geriatric Disorders, Xiangya Hospital, Central South University, Changsha, China; ^3^ Xiangya School of Medicine, Central South University, Changsha, China

**Keywords:** multiple sclerosis, demyelinating disease of the central nervous system, transcranial magnetic stimulation, non-invasive brain stimulation, theta burst stimulation

## Abstract

Multiple sclerosis (MS) is a common chronic, autoimmune-mediated inflammatory and neurodegenerative disease of the central nervous system. The treatment of MS has enormous progress with disease-modifying drugs, but the complexity of the disease course and the clinical symptoms of MS requires personalized treatment and disease management, including non-pharmacological treatment. Transcranial magnetic stimulation (TMS) is a painless and non-invasive brain stimulation technique, which has been widely used in neurological diseases. In this review, we mainly focus on the progress of physiological assessment and treatment of TMS in MS.

## 1 Introduction

Multiple sclerosis (MS), a common type of demyelinating disease of the central nervous system (CNS), is an autoimmune-mediated neurodegenerative disease mainly characterized by inflammatory demyelinating lesions (1). Common symptoms of MS include spasticity, fatigue, pain, movement disorders, cognitive impairment, *etc.*, which have a serious impact on the patient’s quality of life and life expectancy. MS has become the most common cause of nerve dysfunction, in addition to traumatic brain injury, in young adults (2). The effect of drug therapies on disability progression in MS is still unsatisfactory (3). Comprehensive treatments, such as combining transcranial magnetic stimulation (TMS) and other non-drug therapies, are thought to be the therapeutic directions for MS.

TMS is a common type of non-invasive brain stimulation (NIBS). Since its introduction in 1985 by Baker et al. (4), it has developed rapidly and become one of the four major brain technologies in the 21st century. Nowadays, TMS has been widely used for depression (5), neuropathic pain (6), Parkinson’s disease (7), ischemic stroke (8), amyotrophic lateral sclerosis (9), addiction, and CNS degenerative diseases (10–12). There are more and more studies on the application of TMS in MS. The present review here aims to discuss the newest clinical application of TMS in MS.

## 2 Basic principles and classification of TMS

**Figure 1:**
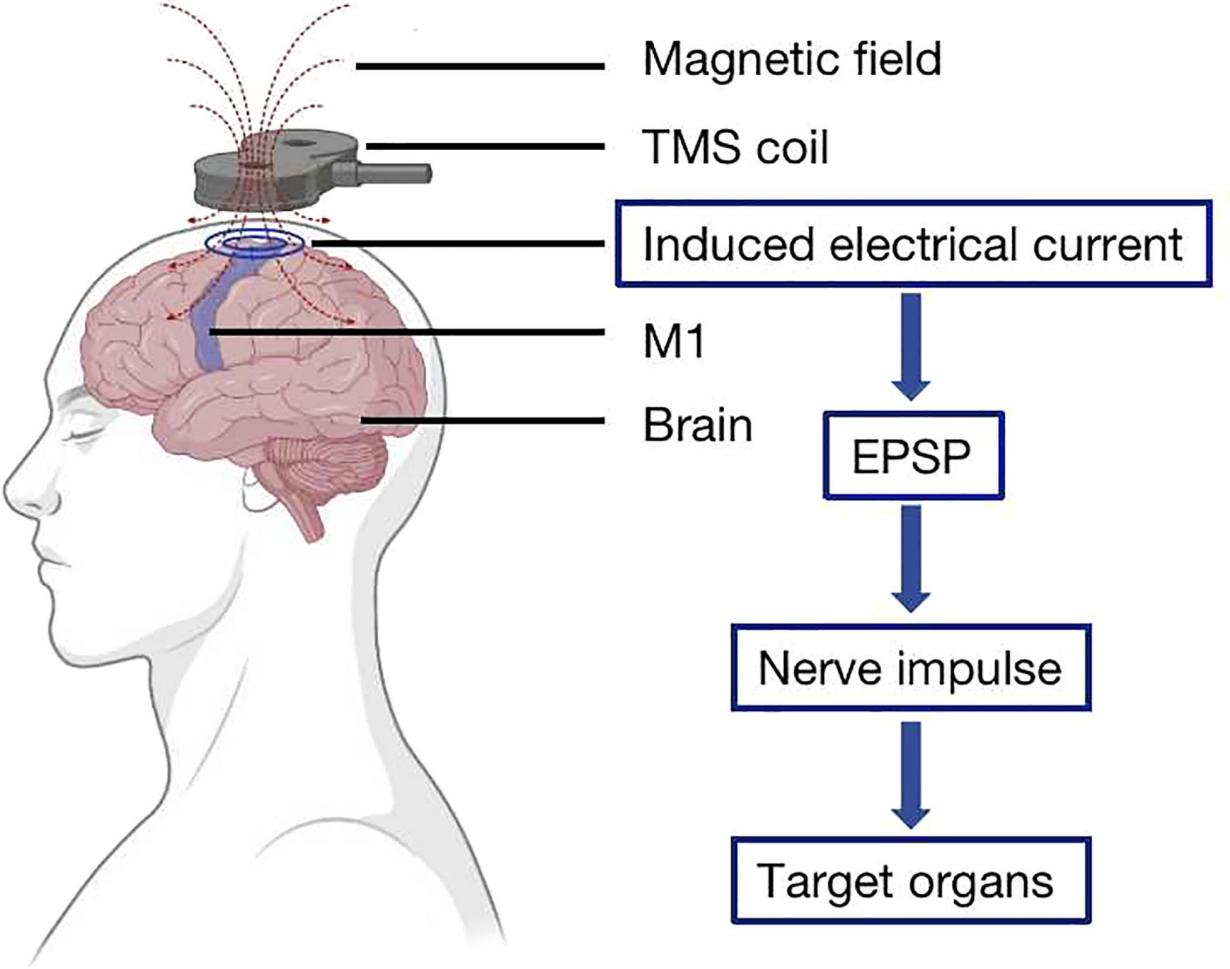
Transcranial magnetic stimulation (TMS) principle. The TMS coil is placed on the surface of the cerebral cortex, and the magnetic field that it generates induces an electrical current in the interneurons located on the corresponding area of the brain, causing the neurons to produce excitatory postsynaptic potential, which generates nerve impulses to the governing organs.

TMS acts on the CNS by a pulsed magnetic field. The TMS device is composed of one or two coils, and the coil is placed on the surface of the corresponding brain region (4). The pulsed magnetic field generated by the coil can induce electrical currents in the interneurons located in the corresponding area of the brain, causing the neurons to produce excitatory postsynaptic potential, which generates nerve impulses. These impulses travel down the axon to the governing organs and perform relevant physiological functions, such as improving the motor function of the controlled muscles (13) (**Figure 1**).

To date, we still know little about the specific cellular mechanism underlying neuromodulation on human brain activity induced by TMS. Some researchers have explored the relevant possible mechanism at the cellular level after applying TMS (14, 15). Short-term TMS pulses lead to the instantaneous inflow of sodium current into cortical neurons and the induction of action potential. Repeated TMS pulses over a long period lead to an increase in steady-state current in depolarized neurons, which subsequently activates L-type calcium channels and postsynaptic N-methyl-D-aspartic acid receptors, resulting in changes of postsynaptic receptor recruitment and activity that affect the long-term plasticity of cortical circuits (15).

**Figure 2:**
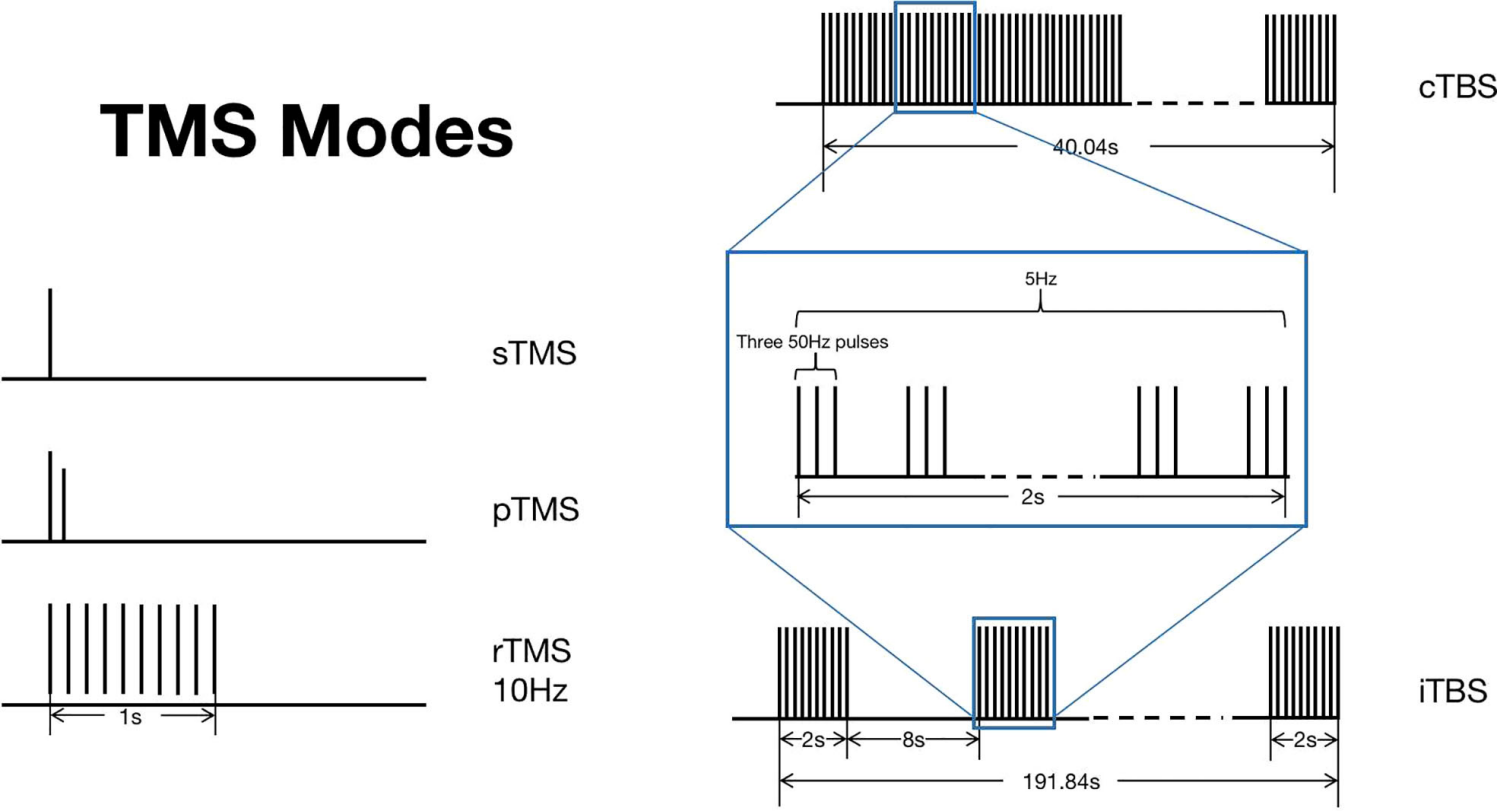
Transcranial magnetic stimulation (TMS) modes. TMS mainly has three stimulation modes, namely sTMS, pTMS, and rTMS. TBS is a new pattern of rTMS, which can be divided into two types: cTBS and iTBS. This figure shows the different stimulation patterns of TMS. .

The TMS technology has many unique advantages—for instance, by changing the position of the coil, TMS can easily stimulate different brain regions, and the TMS evoked potential (TEP) is stable and reliable (16). TMS mainly has three stimulation modes, namely single-pulse TMS (sTMS), paired-pulse TMS (pTMS), and repetitive TMS (rTMS) (17) (**Figure 2**). Among them, sTMS and pTMS are used to explore brain function, while rTMS is used for the treatment of diseases.

rTMS includes both the low-frequency form (<1 Hz) and the high-frequency form (>5 Hz). rTMS can stimulate the corresponding parts of the cerebral cortex and alter the excitability of the resting cortex (17) as well as the functional activity of neurons and the transmission of neurotransmitters between neurons (18). Cortical activity may increase or decrease depending on the frequency and the parameters when multiple pulses are repeatedly applied (19). High-frequency rTMS (HF-rTMS) can increase the cortical excitability, and its effect on improving motor symptoms is mainly due to the increase in the excitability of the stimulated nerves (11, 13). On the contrary, low-frequency rTMS has an inhibitory effect because low-frequency rTMS can reduce either brain metabolism or neuronal activity (20). In addition, the biological effects of rTMS stimulation can persist for a long time after the stimulation (21), which is also different from the temporal effect of sTMS. In general, rTMS can deeply stimulate the brain, maintain a balance between excitatory and inhibitory cortical neurons, regulate the activity of neurocytes and effectively control the excitatory and inhibitory states, regulate the cerebral cortex function, and improve the clinical symptoms.

Theta burst stimulation (TBS) is a new pattern of rTMS, whose principle is similar to rTMS, but it can achieve lasting effect on patients through a shorter stimulation period (22, 23). TBS can be divided into two types: intermittent TBS (iTBS), which belongs to the excitatory mode, and continuous TBS (cTBS), which belongs to the inhibitory mode (22).

## 3 Physical assessment of TMS in MS

TMS can be used to assess cortical excitability and connectivity. Those not only provide essential diagnosis elements of MS but also monitor treatment-induced neuronal changes. Numerous studies had shown that TMS can be a surrogate marker for MS, such as testing disability in MS. As mentioned earlier, sTMS and pTMS are commonly used in the diagnosis of MS. The parameters that can be detected by sTMS include resting motor threshold (RMT), motor evoked potential (MEP), central motor conduction time (CMCT), and so on. Increased RMT and prolonged MEP latencies and CMCT are associated with MS disability. What is more, pTMS can also test the functional connectivity of cortical neuronal populations and pyramidal cells. pTMS refers to a stimulus in which two TMS pulses occur in pairs at a specific interval of time [which is called interstimulus interval (ISI)]. The first pulse of pTMS is called the conditioned TMS (CS), and the second one is called the test TMS (TS). A subthreshold CS followed by a suprathreshold TS is the most commonly used technique (24). When the ISI is short enough (1–5 ms), TS will be in the refractory period of CS-induced potential. The amplitude of TS-induced MEP will decrease, that is, short-interval intracortical inhibition (SICI). When the ISI is 7–30 ms, TS will be in the supernormal period of CS-induced potential; then, the amplitude of TS-induced MEP will increase, that is, intracortical facilitation (ICF). At about 50–200 ms for ISIs, with the same intensity stimulation, a suprathreshold CS followed by TS will decrease the test MEP amplitude compared with the TS alone, which is called long-interval intracortical inhibition (LICI) (24). Intracortical synaptic transmission can be measured by pTMS through intracortical inhibition and ICF. In a study of exploring pTMS as an indicator of disability in MS, researchers found that disability was associated with ICF measures and MEP latency. The short-interval intracortical facilitation (SICF) and MEP latency could be reliable markers of MS disability and be used for the follow-up of disease progression (25).

## 4 Treatment of TMS in MS

Nowadays, TMS has become a widely accepted diagnostic method for MS, and many studies have shown that TMS is also a potential treatment for MS (26). TMS can change the course of MS and improve the symptoms, including spasticity, fatigue, pain, cognitive impairment, *etc.* MS treatment is extremely pharmaceutically dependent and has many limitations. Therefore, the search for new treatments is particularly important, and TMS is an additional way to treat MS. TMS has outstanding features compared with the existing drug treatment methods in many aspects, especially with fewer side effects. What is more, its safety has been confirmed by many related studies (16, 27). Thus, TMS is thought to be a promising new method for the treatment of MS.

### 4.1 Pathogenesis and etiological treatment of MS

MS is a complex autoimmune disease of the CNS affecting more than 2 million people worldwide (28). The pathological conception of MS has been established for a long time, characterized by demyelination and axonal or neuronal loss (29). In the course of the disease, chronic inflammation occurs in the brain, spinal cord, and optic nerves, causing demyelinated plaques, axonal injury, and neuronal loss in the white and gray matter, respectively (30). Demyelination of axons leads to impaired axon conduction, which causes electrical signals to not transmit quickly and efficiently. The axonal and neuronal loss leads to a reduced brain volume, which is commonly known as brain atrophy (29). Axonal and neuronal loss results in the clinical disability of MS patients. The mechanisms involved in the pathogenesis of MS have not been fully elucidated (31). It is now generally accepted that the abnormal immune response, consisting of activated immune cells including both T and B cells, against CNS antigens is probably the major driver of MS pathogenesis (32). Such immune responses trigger inflammatory reactions, oxidative stress, neuronal energy deficit, loss of myelin trophic support, *etc.* All these effects would result in pathological changes such as demyelination and axonal or neuronal loss. Our subsequent discussion of TMS in the treatment of MS will also be relevant. Improvement of nerve conduction function and anti-inflammatory and antioxidant roles is the key to the treatment of MS.

The improvement of nerve conduction function depends on the enhancement of remyelination. It has been found that the application of TMS in the lesion area can promote the remyelination of neurons with demyelinating lesions by activating axonal fibers and neurons and increasing the number of oligodendrocytes (33). The oligodendrocyte, which is closely related to the maintenance of the normal physiological function of myelin, can synthesize myelin to maintain the rapid conduction of action potential across the axon and provide nutrition for axons to meet the large amount of energy required for axon transport (34). Therefore, the effect of TMS on oligodendrocytes is an important mechanism for inducing myelin sheath regeneration. Experimental autoimmune encephalomyelitis (EAE) is an ideal animal model for human MS that can be immunologically induced by several myelin antigens and is widely used to better understand the pathophysiological process and treatment of diseases (35). Researchers have found that TMS can decrease the expression of glial fibrillary acidic protein and Ki67 while enhancing the expression of brain-derived neurotrophic factor and glial cell-derived neurotrophic factor in EAE (36). At the same time, the survival rate of nerve cells can be increased, and the migration of astrocytes can be promoted. Thus, the damage process of CNS can be delayed, and neural protection and remyelination can be promoted (36). Another study using EAE models to investigate the effects of TBS also obtained similar results, suggesting that TBS has a significant therapeutic effect on EAE animal models (37). TBS can alleviate reactive gliosis and promote myelin regeneration, thereby notably improving the neurological symptoms (37). In addition, neuronal activity is also thought to stimulate myelin regeneration (34), so the neuronal activation of TMS may also be a potential mechanism for inducing remyelination.

In terms of inflammation, neuroinflammation mediated by astrocytes and microglia induced by pro-inflammatory mediators is an important pathological basis of MS. In the neuroinflammatory processes of MS, astrocytes grow and become active, promoting the development of inflammation, phagocytosis, production of inflammatory mediators (such as nitric oxide), and antigen presentation, thus resulting in oxidative damage, cellular dysfunction, progressive axonal loss, and neuronal degeneration (38).

Existing studies have found that TMS can play a role in the neuroinflammatory process. Researchers showed that cTBS acting on the EAE model (animal model of MS as mentioned earlier) can downregulate the pro-inflammatory mediator interleukin-1β (IL-1β) and upregulate the anti-inflammatory cytokine interleukin-10 (IL-10), thus playing an anti-inflammatory role. At the same time, cTBS can effectively improve the changes of adenosine signal transduction and weaken the reaction state of microglia and astrocytes in the EAE model, showing a strong potential for protection and repair in the treatment of the model (39). What is more, inflammatory injury in patients with MS can lead to abnormal cortical plasticity. Studies have shown that the cortical plasticity and metaplasticity of MS patients have significantly changed compared with normal people, and such changes can be improved by rTMS and iTBS, thereby promoting the patients’ recovery (40). However, attention should be paid to the use of individualized TMS for different patients (40).

TMS also plays an antioxidant-like role in the treatment process, which can enhance the antioxidant system and reduce cellular oxidative stress damage. At the same time, it can inhibit the apoptosis pathway of mitochondria, reduce the apoptosis of nerve cells, and play a neuroprotective role (41). Aguera et al. presented the case of a woman diagnosed with MS more than a decade earlier and who was not responding to conventional medications (27). In view of this, they decided to try a treatment with rTMS for 1 year. They found that oxidative stress mechanisms had been involved in the etiology and pathogenesis of MS and that the severity of the disease was also related to the intensity of that stress. What is more, rTMS administration could reduce oxidative stress by acting like an antioxidant and exert an objectifiable clinical improvement on MS. In the treatment of the myelin oligodendrocyte glycoprotein (MOG)-induced MS disease model, the researchers compared TMS with three other commonly used drug treatment methods, and they found that TMS can significantly reduce the pathological changes caused by MOG and that its effect is superior to the current clinically common drug treatment such as dexamethasone (42). In the above-mentioned experiment, TMS was observed to decrease the lipid peroxidation products and carbonylated proteins and increase the reduced glutathione/oxidized glutathione ratio, which can well reflect the redox state of cells. Thus, it can be inferred that, at least partially, the effect of TMS application is due to its antioxidant effect (42). Based on existing studies, other scholars have found that TMS can induce the increase in nuclear factor erythroid-2-related factor 2, which is an important factor in inducing the body’s antioxidant response, and lead to the increase in antioxidant enzyme expression, which is one of the possible mechanisms of TMS to exert an antioxidant effect (43). As noted earlier, oxidative damage plays an important role in the pathogenesis of MS, and we have introduced the possible mechanisms revealed by existing studies. There are many other possible potential mechanisms—for example, microglia activation mediated by translocator protein may be also regulated by TMS. The above-mentioned studies all demonstrated that TMS is a potentially effective treatment for MS.

### 4.2 Symptomatic treatment

#### 4.2.1 Spasticity

During the course of MS, about 90% of the patients were reported to have spasticity, which was commonly believed to arise from the stretch reflex hyperexcitability (44, 45), and the exaggerated activation of the stretch reflex is related to an imbalance between the inhibitory and excitatory states of the corticospinal tract, secondary to demyelination of the brain and spinal cord (45). TMS can affect synaptic plasticity, enhance neuronal activity, increase corticospinal excitability, and achieve the purpose of treating spasticity (46, 47). Specifically, after iTBS or rTMS, the MEP amplitude of the stimulated hemisphere increases, resulting in the reduction of the functional connection of the stimulated primary motor cortex (M1), which, in turn, enhances the excitability of the corticospinal tract so as to improve the spastic state (44, 45). It appears that functional reorganization of the M1 may be the basis for the effect of iTBS on MS spasticity (44). Centonze et al. first proposed in 2007 that TMS can ameliorate spasticity in MS (21). They applied rTMS of M1 to MS patients with lower limb spasticity for 2 weeks, and a significant improvement of lower limb spasticity could be observed. What is more, the improvement in symptoms continued up to 7 days after the last treatment. Mori et al. investigated the effects of iTBS in modulating lower limb spasticity in MS patients and found that patients treated with real iTBS had a significant relief of spasticity after 1 week of stimulation, and it continued for another 2 weeks after the end of the stimulation protocol (45). Recently, there are more and more studies on the effects of HF-rTMS and iTBS on relieving spasticity. Korzhova et al. analyzed the results of HF-rTMS and iTBS in the treatment of MS and proved that HF-rTMS (20 Hz) and iTBS have significant effects in the treatment of MS patients with spasticity (48). HF-rTMS not only can improve the symptoms of spasticity but also can reduce the level of pain and fatigue; meanwhile, iTBS can only improve spasticity, but it is the superior one in terms of duration. The retention period of the effect after iTBS treatment is longer than that of HF-rTMS. Therefore, these two methods have their respective advantages in the treatment of MS, and the most appropriate TMS treatment mode should be selected according to the specific situation of the patients. In the evidence-based guidelines for TMS, a review of the 2014–2018 studies on TMS for MS concluded that iTBS relieves spasticity in MS and that iTBS targeting M1 of the legs is rated B (possibly effective) for the treatment of lower limb spasticity in MS patients (11). In terms of treatment methods, the strategies for the TMS treatment of MS spasticity are different in the reported studies (49). Most of the iTBS cases stimulate the M1 area as the most common spot for spasticity of patients with MS (50). Nowadays, more and more studies suggest that TMS therapy should be individualized to determine the most appropriate stimulation protocol for each patient with MS.

#### 4.2.2 Fatigue

Fatigue is one of the most common symptoms in MS and occurs in up to 80% of patients (51). The pathophysiology of fatigue is complex, involving both peripheral mechanisms (such as failure to sustain the force of muscle contraction) and central mechanisms, but “central” abnormalities play a major role in MS (52). Central fatigue appears to be caused by the dysfunction of complex circuits involving the cerebral cortex, the thalamus, and the basal ganglia (53). The mechanism by which TMS improves fatigue is not fully understood and might depend on cortico-cortical and cortico-subcortical mechanisms (47). Various related hypotheses have been presented, including an increase in the spinal drive from M1, modulation of premotor areas, promotion of cortical connectivity, and neuroplasticity through long-term potentiation or long-term depression of synaptic transmission (54). Gaede et al. evaluated the safety and efficacy of deep rTMS in MS fatigue and got a promising result (55). They applied specific H-coils to the left prefrontal cortex and bilateral M1 and found that fatigue improved significantly after stimulation of the M1 area (55). Apart from rTMS, one iTBS protocol applied to the M1 leg area showed that iTBS combined with exercise therapy can significantly ameliorate spasticity and fatigue, while iTBS alone can only reduce spasticity, without having relief from fatigue (56). Another study also showed that iTBS can improve fatigue in MS patients (57). However, due to the limited number and small scale of relevant studies, these results should be considered with caution. More research in related fields is needed to support more reliable conclusions.

#### 4.2.3 Pain

Pain, a common symptom of MS, affects about 70% of the patients, which has a significant impact on the patients’ quality of life and leads to fatigue, depression, and other symptoms (58, 59). Neuropathic pain, nociceptive pain, and headache are common types of MS pain, with headache being the most common, accounting for about 43% (60). To date, the pathophysiological mechanisms of pain in MS are poorly defined. Researchers proposed pain classifications in MS according to pathophysiology, and they agreed that the most common neuropathic pain was a continuous burning sensation in the lower limbs (61). MS pain is considered to be a central pain caused by demyelination of the spinothalamic pathway and areas associated with pain perception. In addition, some researchers believe that pain is closely related to spasticity. Another study on pain in MS demonstrated that gender, age, and disease duration are associated with pain (62). At present, the treatment of pain in MS is highly dependent on drugs, and the results are not satisfactory (51). Thus, Feinstein et al. argue that a combination of multidisciplinary rehabilitative interventions plus new treatments may be the effective strategy for MS pain (51), and NIBS is one of the most popular.

It has been widely recognized that stimulation of the M1 region can relieve neuropathic pain, which is considered to be associated with an increase in corticospinal excitability (47). Researchers have evaluated the efficacy and adverse reactions of motor cortex stimulation (MCS) in detail and proved that MCS can be a safe and effective treatment for neuropathic pain (63). Numerous studies have reported that TMS is an effective method for pain treatment (64). TMS therapy’s analgesic effects are now believed to be based on its ability to affect neurotransmitter systems in the brain, including associated receptors and second messengers, and promote synaptic plasticity. Experts have evaluated TMS for the treatment of pain and demonstrated the feasibility of TMS in various pain treatments (65). What is more, they pointed out that HF-rTMS performed on the left dorsolateral prefrontal cortex (DLPFC) or M1 can play an analgesic effect on patients with neuropathic pain. In the guidelines of TMS, it is also recommended at the contralateral M1 for unilateral neuropathic pain or at the left DLPFC for diffuse neuropathic pain conditions (65). Currently, FDA has approved sTMS for the prevention and treatment of migraine. There are also many related studies that use HF-rTMS to stimulate the M1 region, and its efficacy in preventing migraine has been recognized. So far, the application of rTMS to stimulate the M1 area to treat the pain is the most commonly used and widely recognized strategy, but other TMS forms are also being actively explored to treat pain, such as TBS and so on. In addition, scientists are also interested in other potential targets besides M1 in pain treatment (66). To conclude, the application of the ultimate goal is to achieve better analgesic effect.

#### 4.2.4 Cognitive impairment

Cognitive impairment is a common symptom of MS and has been estimated to be experienced by up to 65% of the patients (67). It should be noted that working memory and information processing efficiency are always affected in MS patients, thus causing great inconvenience to the patients’ daily life. The relief of these symptoms can significantly improve the patients’ quality of life (68). While the generation of cognitive impairment remains not fully understood, it appears that brain cognitive network dysfunction caused by gray matter lesions and brain atrophy is involved. Relevant studies have shown that the use of NIBS techniques such as TMS can play a role in neuromodulation and cognitive recovery, which may promote the rehabilitation of MS patients with cognitive impairment (69). A meta-analysis summarizing several relevant studies in recent years showed that TMS can improve the patients’ cognitive function, especially in working memory, and this improvement is more significant in elderly patients (70). Hulst et al. researched the effect of rTMS on the working memory performance of MS patients by using n-back task evaluation and found that the n-back task accuracy in MS patients improved after rTMS (71). Compared with the healthy controls, the MS patients showed higher task-related frontal activation, which do not have an after-working effect. It appears that rTMS can change the efficiency of the bilateral frontoparietal neural network in MS patients and transfer the brain function of patients to a healthy state. In terms of specific methods of stimulation, Guse et al. provided a systematic review of HF-rTMS studies and found that rTMS (10, 15, or 20 Hz), which was applied over the left DLPFC, is most likely to cause a significant cognitive improvement, within a range of 10–15 successive sessions and an individual motor threshold of 80–110% (72). In addition, correct positioning of the coil is also very important, and functional magnetic resonance imaging-guided TMS neuronavigation targeting the stimulus site is considered to be an effective strategy for achieving correct coil localization, leading to stronger TMS effects and inducing a long-lasting cognitive improvement (73).

As mentioned above, various studies on TMS have identified that it can improve the symptoms of spasticity, fatigue, pain, cognitive impairment, and so on, among which there are more studies on spasticity and fatigue, and the conclusions are more accurate. Overall, the number of studies remains small, and the level of evidence is not conclusive, especially in pain and cognitive impairment. In addition, most of the patients in the existing studies were relapsing–remitting MS. Therefore, we still need to explore whether TMS is beneficial for patients with other forms of MS.

## 5 Conclusion

Exploration of a therapeutic strategy for MS has always been a research hotspot. Improving the clinical symptoms of MS can obviously relieve the pain of patients and improve their quality of life. TMS has been widely recognized for its role in MS. TMS, a painless and non-invasive treatment method, is expected to be used in the clinical treatment of MS. At present, TMS treatment for MS patients is mostly a trial or palliative treatment method, which has not been widely used in clinical treatment. Therefore, in order to widely apply it to the clinical treatment of patients with MS, clinical researchers need to conduct more relevant randomized controlled trials to confirm the therapeutic effect and safety of TMS and study its specific treatment scheme individually so as to strive for a safe and effective systematic treatment scheme as soon as possible. In addition, whether stimulation patterns recommended by existing guidelines are truly effective for all MS patients, whether there is a more optimized TMS protocol, and whether TMS is effective for other MS symptoms beyond existing studies are all worth considering. In conclusion, TMS has been widely recognized as a safe and non-invasive clinical treatment method that is expected to be widely used in the clinical treatment of MS in the future.

## Author contributions

XZ and JL defined the scope of the review. KL consulted related data and wrote this manuscript. QH, JL, XZ, and KL contributed to the revision of the manuscript. All authors contributed to the article and approved the submitted version.

## Conflict of interest

The authors declare that the research was conducted in the absence of any commercial or financial relationships that could be construed as a potential conflict of interest.

## Publisher’s note

All claims expressed in this article are solely those of the authors and do not necessarily represent those of their affiliated organizations, or those of the publisher, the editors and the reviewers. Any product that may be evaluated in this article, or claim that may be made by its manufacturer, is not guaranteed or endorsed by the publisher.
