# Supplementary material for: Clinical application of transcranial magnetic stimulation in multiple sclerosis
Source: Front Immunol. 2022 Sep 5;13:902658. doi: 10.3389/fimmu.2022.902658 (PMC9483183; doi:10.3389/fimmu.2022.902658)
Supplement: Supplementary file 3 [file Table_3.docx]

**Table1 Common TMS parameters**

|  | Definition | Changes in MS vs Healthy* |
| --- | --- | --- |
| RMT | The minimum stimulus intensity required to induce a target muscle (usually abductor brevis) MEP  with a amplitude more than 50μV on at least 5 times out of 10 times stimulations. | Increase |
| MEP | The action potentials recorded in the contralateral target muscle by stimulating the motor cortex | prolonged MEP latencies |
| CMCT | The difference between the latency of MEPs and peripheral conduction time | Increase |
| ICI | TS induced decreased MEP amplitude | a reduction in SICI and an increase in LICI |
| ICF | TS induced increased MEP amplitude | a reduction in SICF |

* Indicator changes in MS patients versus healthy persons.

RMT: resting motor threshold; MEP: motor evoked potential; CMCT: central motor conduction time; ICI: intracortical inhibition; ICF: intracortical facilitation; SICI: short interval intracortical inhibition; LICI: long interval intracortical inhibition; SICF: short interval intracortical facilitation

**Table2 Symptom frequency in MS**

|  | Frequency |
| --- | --- |
| Spasticity | 90%39 |
| Fatigue | 80%47 |
| Pain | 70%54 |
| Cognitive impairment | 65%63 |

**Table3 TMS results in the treatment of multiple sclerosis symptoms**

| Reference | Patients | TMS mode | TMS method | Targeted brain region | Reaults | retention period post-stimulation | clinical scales | study type |
| --- | --- | --- | --- | --- | --- | --- | --- | --- |
| Spasticity |  |  |  |  |  |  |  |  |
| Centonze et al 43 | 19 RRMS | rTMS | 5Hz rTMS, 10 sessions, 2 week protocol | M1(leg region) | Real rTMS reduce spasticity compared to sham | at least 7 days | The H/M amplitude ratio of the soleus H reflex | double-blind, sham-controlled |
| Mori et al 40 | 20 RRMS | iTBS | iTBS, 10 sessions, 2 week protocol | M1(leg region) | Real iTBS reduce spasticity compared to sham | 2 weeks | The H/M amplitude ratio of the soleus H reﬂex, MAS and a reliable neurophysiological index of spinal excitability | double-blind, sham-controlled |
| Mori et al 52 | 30 RRMS | iTBS | iTBS with exercise, 10 sessions, 2 week protocol | M1 contralateral to the spastic limb | iTBS+exercise reduced spasticity and fatigue, iTBS alone could only reduce spasticity, none of the measured scales showed signiﬁcant changes after sham iTBS plus exercise | - | MAS and MSSS-88 | double-blind, sham-controlled |
| Boutière  et al 39 | 17 MS | iTBS | iTBS, one session/day (13 sessions in total), 13 working days protocol | M1(leg region) | Greater improvement of spasticity in real iTBS group than in sham iTBS group | - | MAS and VAS | double-blind, sham-controlled |
| Korzhova et al 44 | 34 SPMS | HF-rTMS | 20Hz HF-rTMS, 10 sessions, 2 weeks protocol | M1 | HF-rTMS and iTBS significant reduce spasticity, while iTBS has a longer-lasting effect and HF-rTMS can reduce pain and fatigue | 2 weeks | MAS, SESS, NAS, MFIS and the pain level scale | double-blind, sham-controlled |
|  |  | iTBS | iTBS, 10 sessions, 2 week protocol | M1 |  | 12 weeks |  |  |
| Fatigue |  |  |  |  |  |  |  |  |
| Gaede et al 51 | 28MS | rTMS | 5Hz rTMS, 3 sessions/week (18 sessions in total), 6 week protocol | left PFC, bilateral M1 | All patients with fatigue showed significant improvement after treatment, and this effect was most pronounced in the moter cortex stimulation group | up to 12 weeks | FSS | prospective, randomized, semi-blinded, sham-con- trolled |
| Mori et al 52 | 30 RRMS | iTBS | iTBS with exercise, 10 sessions, 2 week protocol | M1 contralateral to the spastic limb | iTBS+exercise reduced spasticity and fatigue, iTBS alone could only reduce spasticity, none of the measured scales showed signiﬁcant changes after sham iTBS plus exercise | - | FSS | double-blind, sham-controlled |
| Tramontano et al 53 | 16 MS | c-iTBS | c-iTBS with vestibular rehabilitation (VR), 5 sessions/week (10 sessions in total), 2 week | left and right cerebellum | c-iTBS combined with VR can make MS patients improved in FSS | - | FSS | two-arm, double-blind, randomized controlled |
| Cognitive impairment |  |  |  |  |  |  |  |  |
| Hulst et al 67 | 17 MS, 11 HC | rTMS | 10Hz rTMS, Each participant underwent three sessions (baseline, real-rTMS and sham-rTMS), there was a minimum washout period of 2 weeks between sessions 2 and 3. | right DLPFC | TMS can promote cognitive rehabilitation and shift patients' brain function towards the healthy situation | - | n-back task | randomised, single-blind, sham-controlled, cross-over |

HC: healthy control; FSS: Fatigue Severity Scale; H/M amplitude ratio of the soleus H reflex: a reliable neurophysiologic measure of stretch reflex; MAS: Modiﬁed Ashworth Scale; MSSS-88: the 88 items Multiple Sclerosis Spasticity Score questionnaire; VAS: Visual Analogue Scale; SESS: Subjective Evaluating Spasticity Scale; NAS: Numerical Analog Scale; MFIS: Modified Fatigue Impact Scale; the pain level scale: a questionnaire of pain associated with the increased muscle tone
